# *In vitro* activities of licochalcone A against planktonic cells and biofilm of *Enterococcus faecalis*

**DOI:** 10.3389/fmicb.2022.970901

**Published:** 2022-10-21

**Authors:** Xiaoju Liu, Yanpeng Xiong, Yiyi Shi, Xiangbin Deng, Qiwen Deng, Yansong Liu, Zhijian Yu, Duoyun Li, Jinxin Zheng, Peiyu Li

**Affiliations:** ^1^Department of Infectious Diseases and Shenzhen Key Lab of Endogenous Infection, Huazhong University of Science and Technology Union Shenzhen Hospital, Shenzhen, China; ^2^Department of Infectious Diseases and Shenzhen Key Lab of Endogenous Infection, Shenzhen Nanshan People's Hospital and the 6th Affiliated Hospital of Shenzhen University Health Science Center, Shenzhen, China

**Keywords:** licochalcone A, *Enterococcus faecalis*, antibacterial, persister, biofilm

## Abstract

This study aims to evaluate the *in vitro* antibacterial and anti-biofilm activities of licochalcone A on *Enterococcus faecalis* and to investigate the possible target genes of licochalcone A in *E. faecalis*. This study found that licochalcone A had antibacterial activities against *E. faecalis*, with the MIC_50_ and MIC_90_ were 25 μM. Licochalcone A (at 4 × MIC) indicated a rapid bactericidal effect on *E. faecalis* planktonic cells, and killed more *E. faecalis* planktonic cells (at least 3-log_10_ cfu/ml) than vancomycin, linezolid, or ampicillin at the 2, 4, and 6 h of the time-killing test. Licochalcone A (at 10 × MIC) significantly reduced the production of *E. faecalis* persister cells (at least 2-log_10_ cfu/ml) than vancomycin, linezolid, or ampicillin at the 24, 48, 72, and 96 h of the time-killing test. Licochalcone A (at 1/4 × MIC) significantly inhibited the biofilm formation of *E. faecalis*. The RNA levels of biofilm formation-related genes, *agg, esp*, and *srtA*, markedly decreased when the *E. faecalis* isolates were treated with licochalcone A at 1/4 × MIC for 6 h. To explore the possible target genes of licochalcone A in *E. faecalis*, the licochalcone A non-sensitive *E. faecalis* clones were selected *in vitro* by induction of wildtype strains for about 140 days under the pressure of licochalcone A, and mutations in the possible target genes were detected by whole-genome sequencing. This study found that there were 11 nucleotide mutations leading to nonsynonymous mutations of 8 amino acids, and among these amino acid mutations, there were 3 mutations located in transcriptional regulator genes (MarR family transcriptional regulator, TetR family transcriptional regulator, and MerR family transcriptional regulator). In conclusion, this study found that licochalcone A had an antibacterial effect on *E. faecalis*, and significantly inhibited the biofilm formation of *E. faecalis* at subinhibitory concentrations.

## Introduction

*Enterococcus faecalis* is an opportunistic pathogen, but in recent years, *E. faecalis* has caused more and more clinical infections (urinary tract infection, bloodstream infection, wound infection, and so on; García-Solache and Rice, 2019). With the increased cases of *E. faecalis* infections, drug resistance caused by the wide use of antimicrobials is also increasing. What deserves our attention is that with the gradual increase of multidrug-resistant (MDR) *E. faecalis* infections, it has brought new challenges and difficulties to clinical treatment (Pöntinen et al., 2021). Linezolid, an important member of the oxazolidinone class of antibiotics, is one of the first-line drugs for the treatment of MDR *E. faecalis* infections. However, with the increasing use of linezolid, growing cases of linezolid-resistant *E. faecalis* have emerged (Egan et al., 2020). Therefore, now we still need to explore compounds or natural products with antibacterial activities against *E. faecalis* infections.

In addition to the drug resistance problem of *E. faecalis* infections, another issue is that *E. faecalis* has a strong biofilm-forming ability. For example, 100% of *E. faecalis* isolated from bloodstream infections in Britain could form biofilms (Sandoe et al., 2003). *E. faecalis* biofilm is a membrane composed of an extracellular matrix (ECM), proteins, exopolysaccharides (EPSs), and some micromolecules (such as Edna; Mohamed and Huang, 2007). This structure can significantly reduce the sensitivity of bacteria to antimicrobials and escape the attack and phagocytosis of host immune cells, resulting in chronic infection and non-healing (Goh et al., 2017). In addition, *E. faecalis* could produce persisters under the pressure of antimicrobials, leading to poor efficacy of antimicrobials and even treatment failure (Kaviar et al., 2022). Bacteria persisters are resistant to antimicrobials mainly through a complex dormancy mechanism, and the lack of antibacterial activity to inhibit the formation of persisters is an important reason for the poor treatment effect of many antimicrobials (Fisher et al., 2017). Although there are many studies on the inhibition of bacterial biofilm or persisters, there are still no antibacterial drugs with good effects in clinical anti-biofilm or anti-persister treatment. Therefore, the development of new antimicrobials to inhibit the bacteria biofilm formation or persisters is expected to improve the effectiveness of current anti-infection treatment (Del Pozo, 2018).

Licochalcone A (Lic), a flavonoid isolated from the common Chinese medicinal herb *Glycyrrhiza uralensis* Fisch, presents obvious anti-cancer effects and displays broad-spectrum inhibition against UDP-glucuronosyltransferases (UGTs; Tang et al., 2016). Licochalcone A exhibits strong inhibitory effects against UDP-glucuronosyltransferase 1A1, 1A3, 1A4, 1A6, 1A7, 1A9, and 2B7 (Xin et al., 2016). Interestingly, previous studies have found that licochalcone A had bactericidal activity against Gram-positive *Streptococcus suis* and *Staphylococcus aureus*, and even has a certain inhibitory effect on the biofilm of *S. aureus* (Hao et al., 2013; Shen et al., 2015). In recent years, Grenier et al. also reported that nisin in combination with licochalcone A efficiently inhibited the growth of *E. faecalis*, and even reduced the pre-formed biofilm of *E. faecalis* ATCC 19433 strain (Grenier et al., 2020). However, the alone role and mechanism of licochalcone A against *E. faecalis* are still unclear. Thus, this study aims to evaluate the antibacterial and anti-biofilm activities of licochalcone A against *E. faecalis* and to explore the possible target genes of licochalcone A in *E. faecalis*.

## Materials and methods

### Bacterial isolates and culture conditions

There were 276 *E. faecalis* clinical isolates used in this study, which were collected from Huazhong University of Science and Technology Union Shenzhen Hospital (Grade A, level III Hospital, 1,500 beds) between 1 January 2016 and 31 May 2021. All clinical isolates were identified with a Phoenix 100 automated microbiology system (BD, Franklin Lakes, NJ, USA) and two subcultured generations were re-identified with matrix-assisted laser desorption ionization time-of-flight mass spectrometry (IVD MALDI Biotyper, Germany). The *E. faecalis* ATCC29212 and OG1RF were purchased from American Type Culture Collection (ATCC) and used as reference strains.

*E. faecalis* isolates were grown in tryptic soy broth (TSB) at 37°C with shaking of 180 rpm unless otherwise stated. *E. faecalis* isolates were grown in cation-adjusted Mueller-Hinton broth (CAMHB) at 37°C with shaking for antimicrobial susceptibility test and time-killing test. *E. faecalis* isolates were grown in TSBG (TSB with 0.5% glucose) at 37°C for biofilm assay. CAMHB media were supplemented with 50 mg/L Ca^2+^ for testing of daptomycin in all experiments.

### Chemicals or natural products

Ampicillin sodium (catalog no. HY-B0522A), vancomycin hydrochloride (catalog no. HY-17362), linezolid (catalog no. HY-10394), daptomycin (catalog no. HY-B0108), rifampin (catalog no. HY-B0272), minocycline hydrochloride (catalog no. HY-17412), fosfomycin (catalog no. HY-B1075), and licochalcone A (catalog no. HY-N0372) were purchased from MedChemExpress (Shanghai, China). Gentamicin (catalog no. E003632) and glucose-6-phosphate (catalog no. V900924) were purchased from SIGMA-ALDRICH (Shanghai, China).

### Antimicrobial susceptibility test

The minimum inhibitory concentrations (MICs) for ampicillin, vancomycin, linezolid, daptomycin, rifampin, minocycline, gentamicin, and licochalcone A against *E. faecalis* isolates were determined by the broth macrodilution method in cation-adjusted Mueller-Hinton broth (CAMHB), and the MIC for fosfomycin against *E. faecalis* isolates was measured by the agar dilution method according to the Clinical and Laboratory Standards Institute guidelines (CLSI-M100-S30). All experiments were performed in triplicate.

### Time-killing test

To explore the rapid bactericidal activity of licochalcone A against *E. faecalis* planktonic cells, two *E. faecalis* isolates (16C51, isolated from blood; 16C106, isolated from urine) in logarithmic growth phase were tested and compared with ampicillin, vancomycin, and linezolid. The time-killing test was performed according to our previous research with minor modifications (Zheng et al., 2019): *E. faecalis* isolates were cultivated overnight in CAMHB at 37°C. Overnight cultures were diluted 1:200 in fresh CAMHB and were cultured to the middle logarithmic growth phase (3.5 h) in a 14-ml Polypropylene Round-Bottom Tube in a final volume of 6 ml. Then, licochalcone A, ampicillin, vancomycin, and linezolid were added to the cultures (Final concentration at 4 × MIC), no antimicrobials were used as control, and were further incubated at 37°C with shaking. At the time points of 2, 4, 6, 12, and 24 h, 1 ml aliquots were sampled and washed with 0.9% saline solution. Ten-fold dilutions were then plated on Muller-Hinton agar, and the numbers of CFU were determined. All experiments were performed in triplicate.

The inhibitory activity of licochalcone A on *E. faecalis* persister cells was tested according to previous research (Lee et al., 2019): *E. faecalis* isolates were cultivated overnight in CAMHB at 37°C for 12 h to the stationary growth phase in 14 ml Polypropylene Round-Bottom Tube in a final volume of 6 ml. Then, licochalcone A, ampicillin, vancomycin, and linezolid were added to the overnight cultures (Final concentration at 10 × MIC), no antimicrobials were used as control, and were further incubated at 37°C with shaking. At the time points of 24, 48, 72, 96, and 120 h, 1 ml aliquots were sampled and washed with 0.9% saline solution. Ten-fold dilutions were then plated on Muller-Hinton agar, and the numbers of CFU were determined. All experiments were performed in triplicate.

### Detection of growth curve of *E. Faecalis*

The growth curve of *E. faecalis* isolates was detected by a Finnish bioscreen automatic growth curve analyzer: *E. faecalis* isolates were cultivated overnight in TSB at 37°C. Overnight cultures were diluted 1:200 in fresh TSB and added with sub-MICs of licochalcone A, then the cultures were inoculated into 96-well polystyrene microtiter plates at the amount of 300 μL/well and TSB without licochalcone A was used as untreated control. The optical density at 600(OD_600_) was tested by the Bioscreen C system (Lab Systems Helsinki, Finland) according to the instrument manual to set the procedure. The experiment was recorded for 24 h. Each assay was performed in triplicate at least three times.

### Exploration of the effect of licochalcone A on the biofilm formation of *E. faecalis*

Biofilm biomasses of *E. faecalis* were determined by the crystal violet staining method based on our previous studies with some modifications (Mohamed et al., 2004). After overnight culture, *E. faecalis* was diluted 1:100 with fresh TSBG containing sub-MICs of licochalcone A and inoculated into 96-well polystyrene microtiter plates, and no licochalcone A was used as control. After static incubation for 24 h, the supernatant was removed and plates were gently washed with phosphate-buffered saline (PBS), dried at room temperature, and fixed with methanol for 15 min. Methanol was removed and cells were stained with 0.5% crystal violet for 10 min at room temperature. Crystal violet was dissolved in 95% ethanol and OD_570_ was determined. This experiment was performed in triplicate at least three times.

### Investigation of the effect of licochalcone A on the established biofilm of *E. faecalis*

Overnight cultures of *E. faecalis* were diluted 1:100 with fresh TSBG and inoculated into 96-well polystyrene microtiter plates, after static incubation for 24 h at 37°C (mature biofilms established), the supernatant was removed and plates were washed with 0.9% saline to remove unattached cells. Then, the fresh TSBG containing different concentrations of licochalcone A, or combined with ampicillin, vancomycin, and linezolid was added, and no licochalcone A was used as control. After static incubation for 24 h, plates were washed gently three times with PBS, dried at room temperature, and fixed with methanol for 15 min. Methanol was removed and cells were stained with 0.5% crystal violet for 10 min at room temperature. Crystal violet was dissolved in 95% ethanol and OD_570_ was determined. This experiment was performed in triplicate at least three times.

### Detection of the RNA levels of biofilm formation-related genes of *E. faecalis*

To explore the possible mechanism of licochalcone A inhibiting the biofilm formation of *E. faecalis*, the transcription levels of biofilm formation-related genes of *E. faecalis* were detected by RT-qPCR according to our previous study (Zheng et al., 2020). In brief, overnight cultures of *E. faecalis* isolates (16C51 and 16C106, with the ability to form strong biofilms) were diluted 1:200 with fresh TSBG containing 1/4 × MIC of licochalcone A and inoculated into 25-mL polypropylene round culture dishes, and no licochalcone A was used as control. After static incubation for 6, 12, and 24 h at 37°C, *E. faecalis* biofilms were harvested from the polypropylene round culture dishes using cell scrapers, and total RNA was collected from the planktonic cells and biofilms for RT-qPCR. RT-qPCR was conducted using the SYBR green PCR reagents (SYBR Premix *Ex Taq*; TaKaRa Biotechnology, Dalian, China) in the Mastercycler RealPlex system (Eppendorf AG, Hamburg, Germany). The housekeeping gene *recA* gene was used as an internal reference gene to normalize transcript levels. The untreated control isolates were used as the reference isolates. The primers used for RT-qPCR in this study were listed in Supplementary Table S1. RT-qPCR was performed in triplicate.

### *In vitro* induction of licochalcone A non-sensitive *E. faecalis* isolates with possible mutations

To explore the possible target genes of licochalcone A in *E. faecalis*, the licochalcone A non-sensitive *E. faecalis* isolates with possible mutations were induced and screened *in vitro* according to previous studies (Zheng et al., 2021; Liu et al., 2022). *E. faecalis* isolates (16C51, 16C106) were subcultured serially in TSB containing licochalcone A. The initial inducing concentration of licochalcone A was 1/2 × MIC; the concentration was then increased successively to high concentrations. *E. faecalis* isolates in each concentration of licochalcone A were cultured for three to five passages before being inoculated and passaged to the next generation. *E. faecalis* isolates from the last passage of each concentration of licochalcone A were collected and subcultured on tryptic soy agar plates without licochalcone A for three passages, identified again by matrix-assisted laser desorption ionization time-of-flight mass spectrometry (IVD MALDI Biotyper, Bruker, Bremen, Germany), and the MIC of licochalcone A was measured again. Finally, the licochalcone A non-sensitive *E. faecalis* isolates were kept frozen at −80°C in glycerol containing (35%) TSB.

### Detection of mutations in licochalcone A non-sensitive *E. faecalis* isolates by the whole-genome sequencing

The genomic DNA of two licochalcone A non-sensitive *E. faecalis* isolates (16C51-T1.1 and 16C51-T1.2) was extracted, and a total amount of 1 μg DNA per sample was used as input material for DNA sample preparations. Whole genomes were sequenced in an Illumina HiSeq2500 sequencer according to the previous study (Zheng et al., 2022). Coding genes, repetitive sequences, non-coding RNAs, genomics islands, transposons, prophages, and CRISPR (clustered regularly interspaced short palindromic repeat) sequences were predicted. Gene functions were predicted by referring to the following databases: GO (Gene Ontology), KEGG (Kyoto Encyclopedia of Genes and Genomes), and Swiss-Prot. Genomic alignments between each sample genome and a reference genome (*E. faecalis* OG1RF, GenBank: NC_017316.1) were performed with MUMmer and LASTZ tools. Single nucleotide polymorphisms, insertions, deletions, and structural variation annotations were identified based on inter-sample genomic alignment results by MUMmer and LASTZ.

### Statistical analysis

Data were analyzed by Student's *t*-test. *P*-values < 0.05 were regarded as statistically significant. All data were analyzed in the SPSS software package (version 19.0, Chicago, IL, USA).

## Results

### Antimicrobial susceptibility of licochalcone A against *E. faecalis*

To explore the antibacterial activities of licochalcone A against *E. faecalis*, the MICs of licochalcone A and other antimicrobials against *E. faecalis* were determined, and the susceptibility results were confirmed according to the CLSI-M100-S30. The MICs of licochalcone A against *E. faecalis* reference strains ATCC29212 and OG1RF were detected, and all the MICs were 12.5 μM (equal to 4.23 mg/L). Subsequently, the MICs of licochalcone A against 22 *E. faecalis* clinical isolates (isolated from blood or urine) were measured, of which the MICs against 17 isolates were 25 μM and the MICs against 5 isolates were 12.5 μM (Table 1). Among the 22 *E. faecalis* clinical isolates, there were 3 linezolid non-sensitive isolates, but the MICs of licochalcone A against these isolates did not increase. To further verify the antibacterial activity of licochalcone A against *E. faecalis* clinical isolates, the MICs of licochalcone A against other 254 *E. faecalis* clinical isolates (isolated from urine, sputum, tissue, catheters, pleural effusion, ascites fluid, amniotic fluid, puncture fluid, and cerebrospinal fluid) were determined. This study indicated that the MICs of licochalcone A were 12.5 to 50 μM, and the MIC_50_ and MIC_90_ were 25 μM against these clinical isolates (data not shown).

**Table 1:** Antimicrobial susceptibilities of *E. faecalis* determined by conventional broth macrodilution or agar dilution method.

**Strains**	**MIC (mg/L)**								**MIC**
	**Amp**	**Van**	**Lin**	**Dap**	**Rif**	**Min**	**Gen**	**Fos^a^**	**Lic**
ATCC29212	2	2	2	4	1	4	8	32	12.5 μM (4.23 mg/L)
OG1RF	2	2	2	4	128	1	16	64	12.5 μM (4.23 mg/L)
FB-1	2	1	8	4	8	1	>512	32	25 μM (8.46 mg/L)
16C35	4	1	2	8	8	16	16	64	25 μM (8.46 mg/L)
16C51	2	2	4	8	8	16	8	64	25 μM (8.46 mg/L)
16C54	2	1	2	16	16	8	>512	64	25 μM (8.46 mg/L)
16C68	4	1	2	4	4	16	>512	64	12.5 μM (4.23 mg/L)
16C102	4	1	8	4	8	8	32	32	25 μM (8.46 mg/L)
16C106	2	1	2	8	8	16	>512	64	25 μM (8.46 mg/L)
16C124	1	1	2	4	8	16	8	64	25 μM (8.46 mg/L)
16C125	2	1	2	4	2	16	>512	64	12.5 μM (4.23 mg/L)
16C137	2	0.5	2	8	32	16	>512	64	25 μM (8.46 mg/L)
16C138	2	1	2	8	8	16	>512	32	25 μM (8.46 mg/L)
16C152	2	1	2	2	4	16	>512	64	25 μM (8.46 mg/L)
16C166	2	1	2	8	4	16	16	32	25 μM (8.46 mg/L)
16C168	4	1	2	8	2	16	>512	64	12.5 μM (4.23 mg/L)
16C186	2	0.5	2	8	8	8	16	64	12.5 μM (4.23 mg/L)
16C201	2	1	2	4	8	16	>512	32	25 μM (8.46 mg/L)
16C274	4	1	2	8	16	16	16	64	25 μM (8.46 mg/L)
16C289	2	1	2	2	2	16	8	64	25 μM (8.46 mg/L)
16C350	2	1	2	4	2	16	>512	32	25 μM (8.46 mg/L)
16C353	2	1	2	16	16	16	16	32	25 μM (8.46 mg/L)
16C385	2	0.5	2	4	16	16	>512	64	25 μM (8.46 mg/L)
16C405	2	1	2	4	2	16	>512	64	12.5 μM (4.23 mg/L)

MIC, minimum inhibitory concentration; Amp, Ampicillin; Van, Vancomycin; Lin, Linezolid; Dap, Daptomycin; Rif, Rifampin; Min, Minocycline; Gen, Gentamicin; Fos, Fosfomycin; Lic, licochalcone A; ^a^agar dilution method.

### The rapid bactericidal activity of licochalcone A against *E. Faecalis* planktonic cells

**Figure 1:**
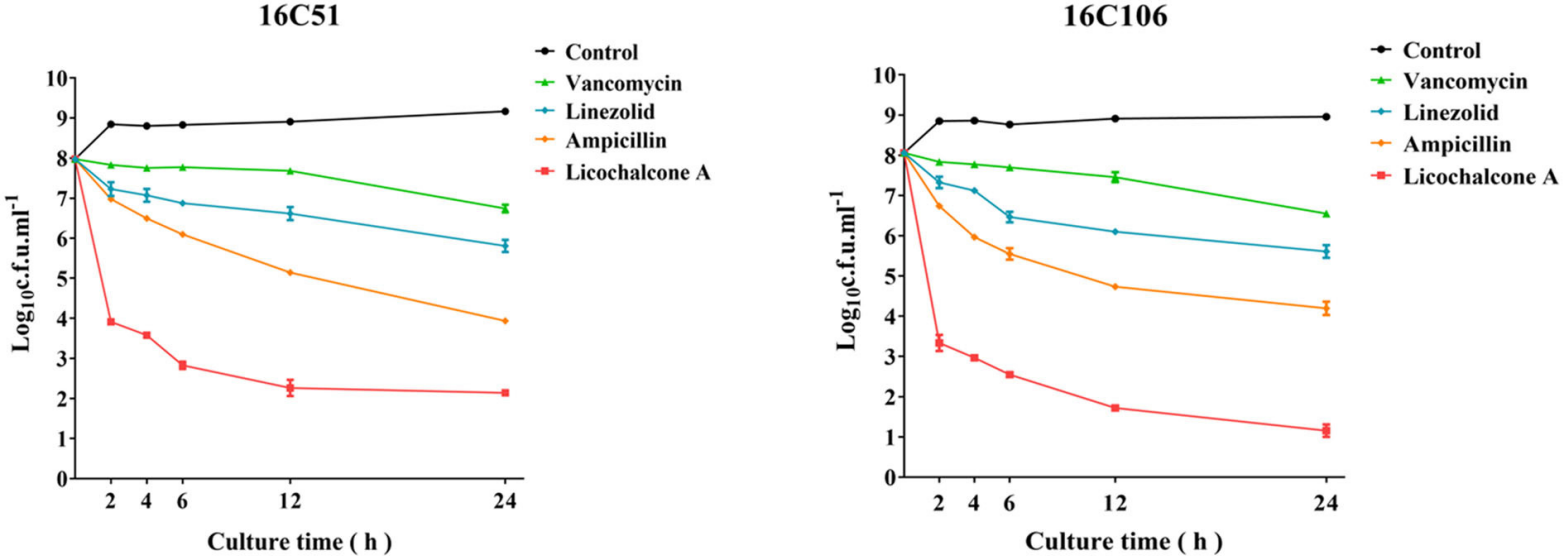
The anti-bacterial effect of licochalcone A on the planktonic cells of the two *E. faecalis* isolates during logarithmic growth was detected by time-killing studies. The data presented were the average of three independent experiments (mean ± SD). Vancomycin, Linezolid, Ampicillin, and licochalcone A were used at 4 × MIC. MIC, minimum inhibitory concentration.

The rapid bactericidal activities of licochalcone A on *E. faecalis* planktonic cells were determined by the time-killing test and compared with vancomycin, linezolid, and ampicillin (all used at 4 × MIC) (Oliva et al., 2014). As Figure 1 indicated, licochalcone A showed a rapid bactericidal effect on *E. faecalis* planktonic cells, and killed more planktonic cells (at least 3-log_10_ cfu/ml) than vancomycin, linezolid, and ampicillin at 2, 4, and 6 h of the time-killing test. The results also showed that licochalcone A killed more *E. faecalis* planktonic cells (at least 2-log_10_ cfu/ml) than vancomycin, linezolid, and ampicillin even at the 12 and 24 h of the time-killing test.

### High concentration of licochalcone A reduced the production of *E. Faecalis* persister cells

**Figure 2:**
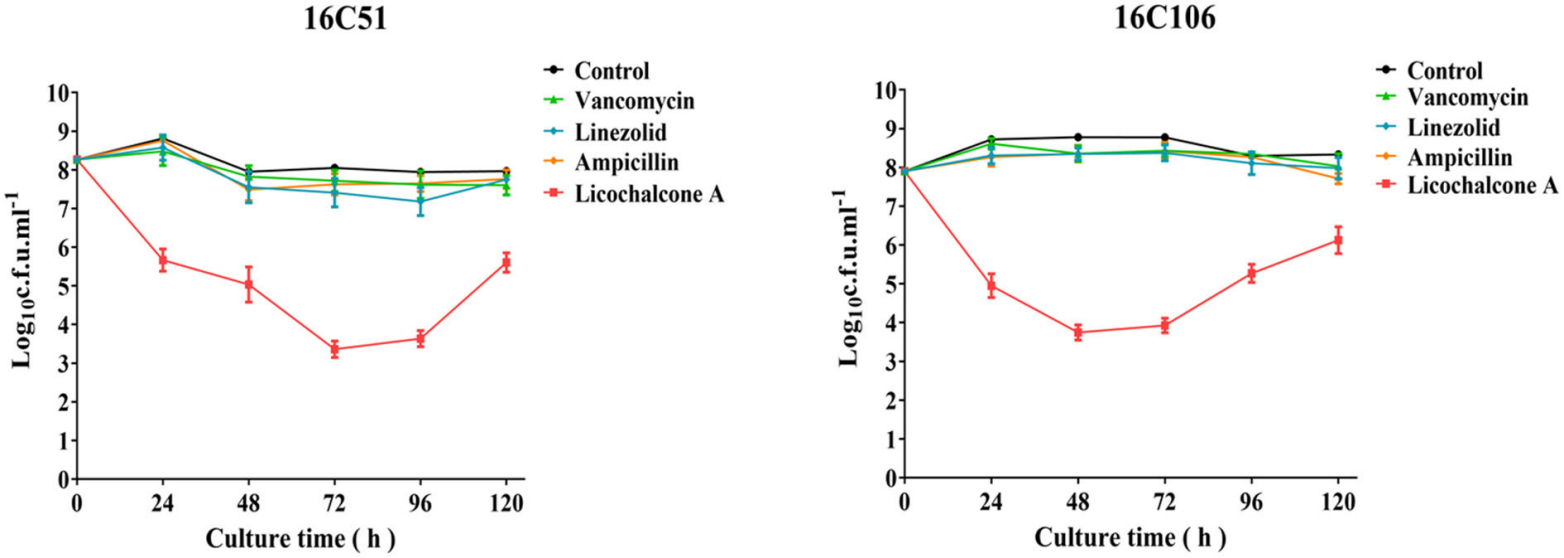
The inhibitory effect of licochalcone A on the persister cells of the two *E. faecalis* isolates during stationary growth was detected by time-killing studies. The data presented were the average of three independent experiments (mean ± SD). Vancomycin, Linezolid, Ampicillin, and licochalcone A were used at 10 × MIC. MIC, minimum inhibitory concentration.

At present, it is also found that bacteria can produce persister cells under the pressure of antimicrobials (Fisher et al., 2017). Therefore, this study explored the inhibitory effect of licochalcone A on the production of *E. faecalis* persister cells at high concentrations, and compared it with vancomycin, linezolid, and ampicillin (all used at 10 × MIC). As Figure 2 demonstrated, licochalcone A significantly reduced the production of *E. faecalis* persister cells (at least 2-log_10_ cfu/ml) than vancomycin, linezolid, and ampicillin at 24, 48, 72, and 96 h of the time-killing test.

### Subinhibitory concentrations of licochalcone A significantly reduce the biofilm formation of *E. faecalis*

**Figure 3:**
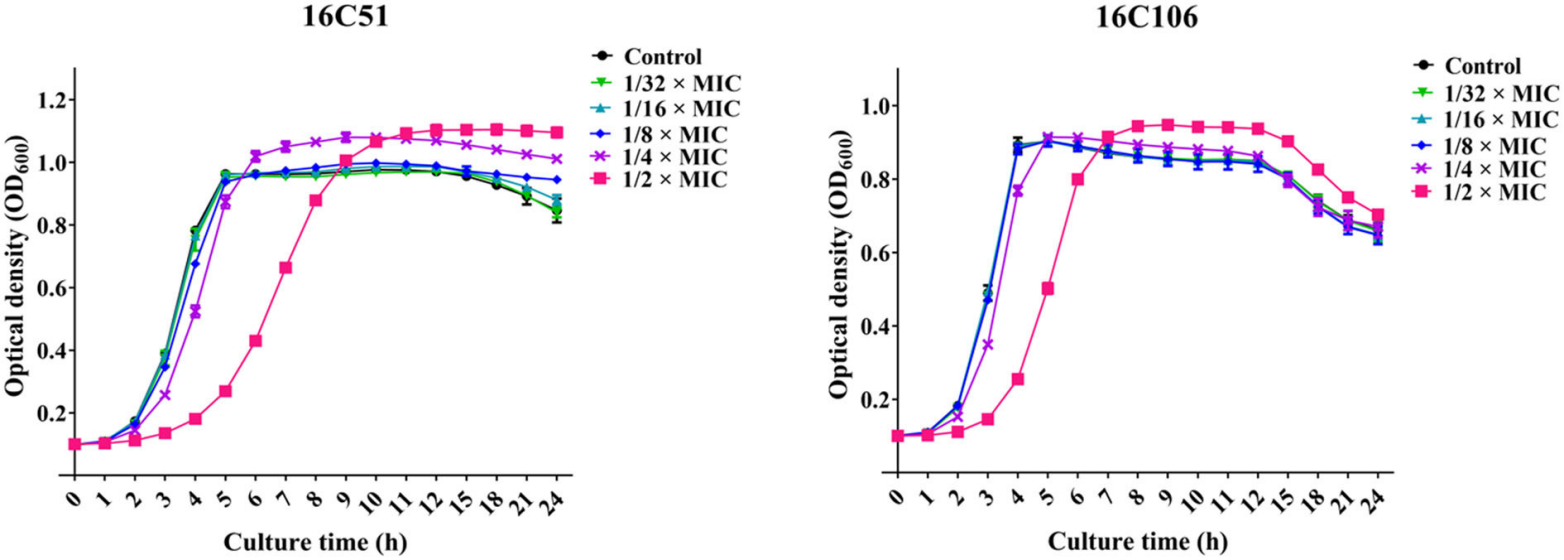
The effect of sub-MIC concentrations of licochalcone A on the growth of the two *E. faecalis* isolates planktonic cells. The growth of *E. faecalis* isolates planktonic cells was determined by optical density at 600 nm (OD_600_). The data presented were the average of three independent experiments (mean ± SD). MIC, minimum inhibitory concentration.

**Figure 4:**
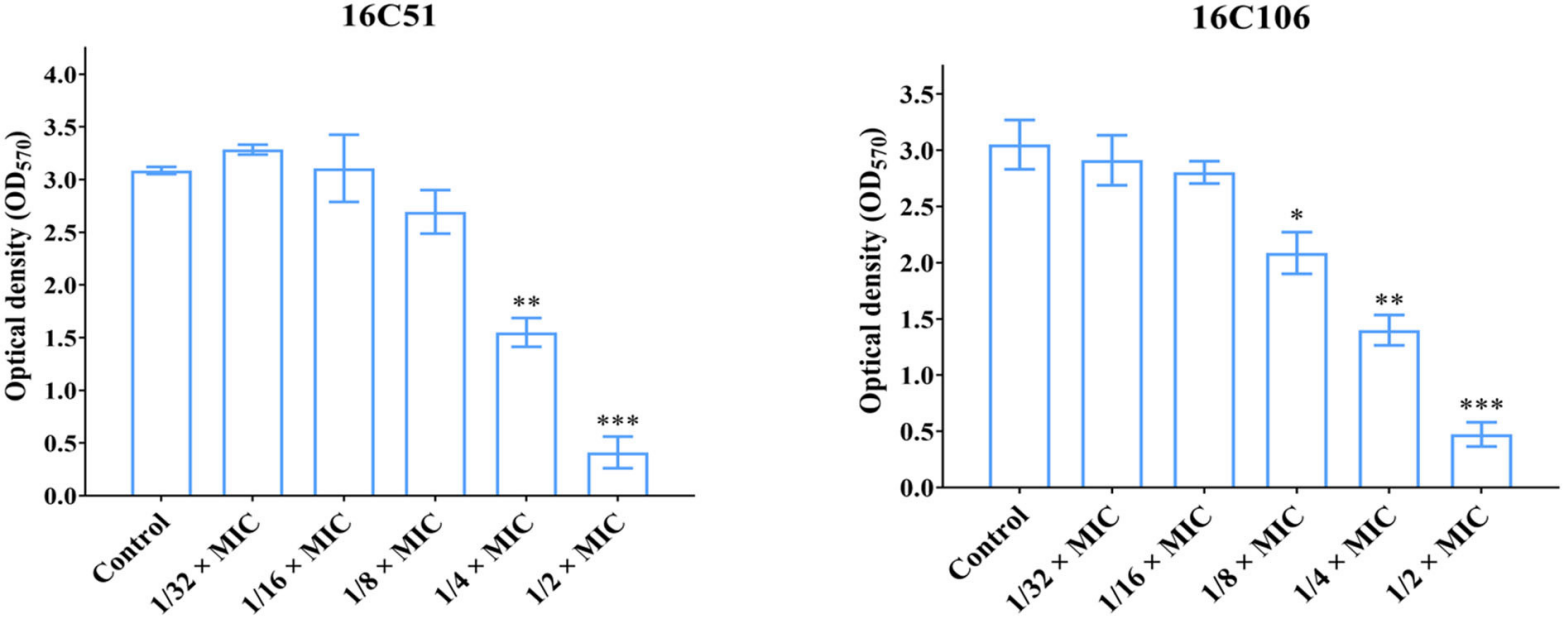
The effect of sub-MIC concentrations of licochalcone A on the biofilm formation of the two *E. faecalis* isolates. The two *E. faecalis* isolates (16C51 and 16C106) were treated with licochalcone A at sub-MIC concentrations for 24 h, and the biofilm biomasses were determined by crystal violet staining. The data presented were the average of three independent experiments (mean ± SD). Compared with control, *P < 0.05; **P < 0.01; ***P < 0.001; (Student's *t*-test). MIC, minimum inhibitory concentration.

**Figure 5:**
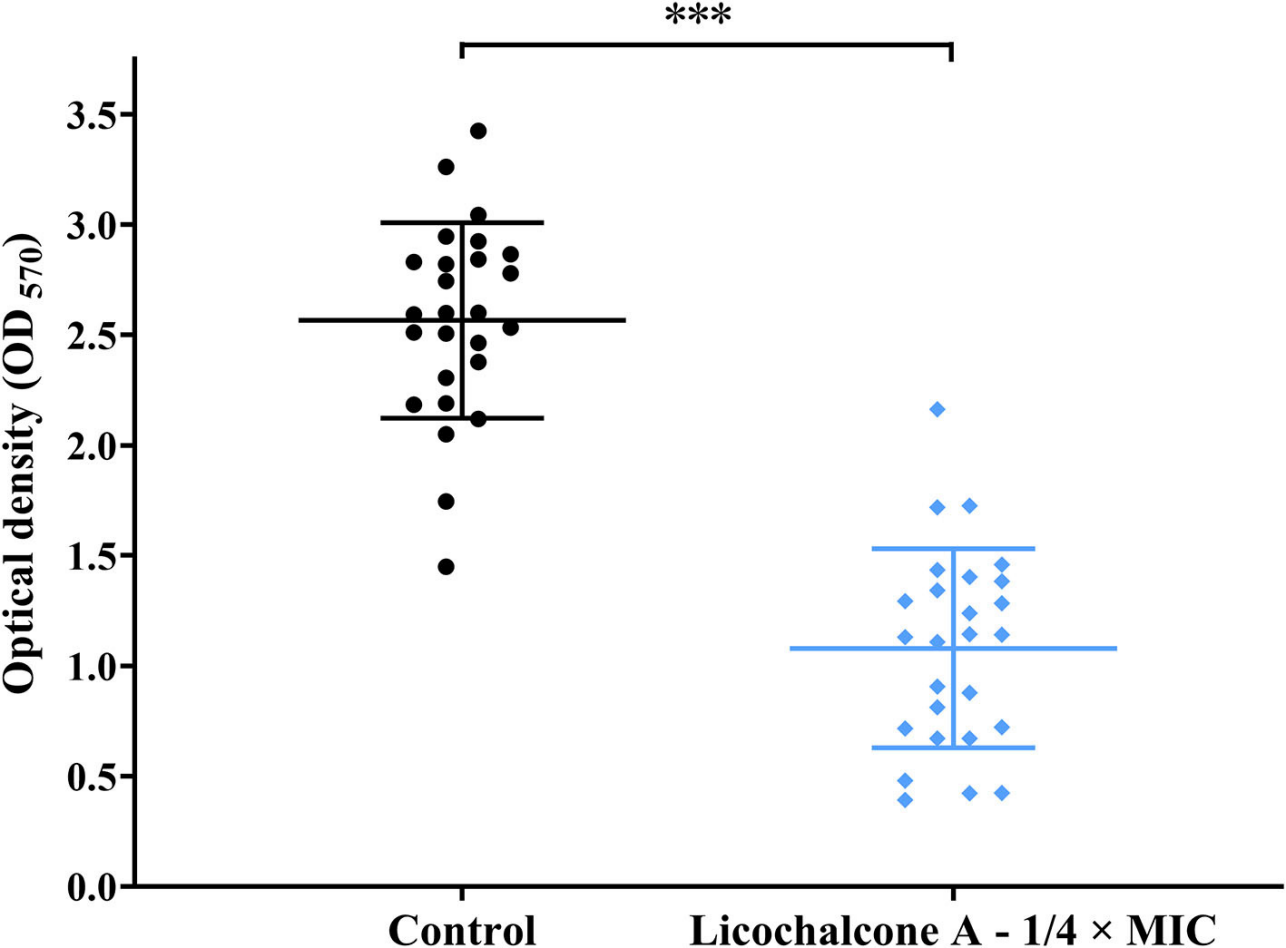
The effect of 1/4 × MIC of licochalcone A on the biofilm formation of the 26 *E. faecalis* isolates. The 26 *E. faecalis* isolates were treated with licochalcone A at 1/4 × MIC for 24 h, and the biofilm biomasses were determined by crystal violet staining. The data presented were the results of three independent experiments, and take the mean value of three independent experiments for each strain for analysis. The results of 26 *E. faecalis* isolates in each group were presented as a mean ± 95% confidence interval. Compared with control, ***P < 0.001; (Student's t-test). MIC, minimum inhibitory concentration.

To study the effect of subinhibitory concentrations of licochalcone A on the biofilm formation of *E. faecalis*, the effect of subinhibitory concentrations of licochalcone A on the growth of *E. faecalis* planktonic cells was determined. This study found that the 1/2 × MIC of licochalcone A inhibited the growth of *E. faecalis* planktonic cells in the logarithmic growth phase, while 1/4 ×, 1/8 ×, 1/16 ×, and 1/32 × MICs had no inhibitory effect (Figure 3). Interestingly, this study indicated that biofilm formation of the two *E. faecalis* isolates (16C51, 16C106) were significantly inhibited by 1/2 × or 1/4 × MICs of licochalcone A (Figure 4). However, the growth of *E. faecalis* planktonic cells was inhibited by the 1/2 × MIC of licochalcone A (Figure 3); thus, licochalcone A at 1/4 × MIC had the capacity of inhibiting biofilm formation of *E. faecalis*, but had no inhibitory effect on the growth of its planktonic cell. This interesting finding was also verified in more *E. faecalis* clinical isolates (Figure 5).

**Figure 6:**
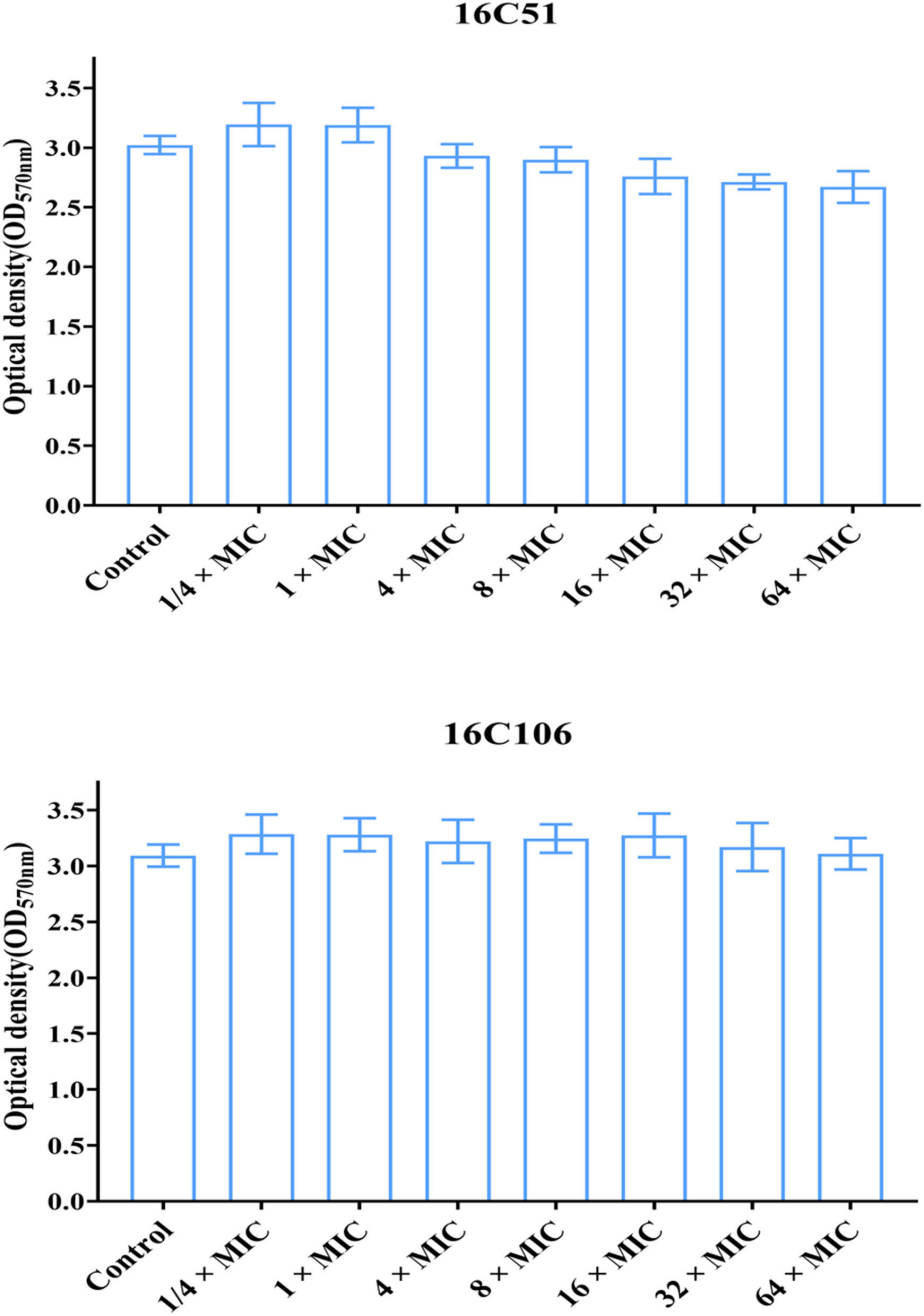
The effect of different concentrations of licochalcone A on the established biofilms of the two *E. faecalis* isolates. The two *E. faecalis* isolates (16C51 and 16C106) formed mature biofilms for 24 h, then treated with licochalcone A at different concentrations for 24 h, and the remaining biofilm biomasses were determined by crystal violet staining. The data presented were the average of three independent experiments (mean ± SD). MIC, minimum inhibitory concentration.

This study also explored the eradicating effect of different concentrations of licochalcone A (from 1/4 × to 64 × MICs) on the established biofilms of *E. faecalis*, but found no effect (Figure 6). This study continued to explore licochalcone A combined with vancomycin, linezolid, or ampicillin (all used at 8 × MIC) to treat the established biofilms of *E. faecalis*, but still found no eradicating effect (Supplementary Figure S1).

### The RNA levels of biofilm formation-related genes of *E. faecalis* were inhibited by licochalcone A

The two *E. faecalis* isolates (16C51, 16C106) were selected for RT-qPCR to determine the RNA levels of 16 biofilm formation-related genes of *E. faecalis* based on previous studies (Tendolkar et al., 2005; Nallapareddy et al., 2006; Guiton et al., 2009; Chavez De Paz et al., 2012; Soares et al., 2014; Dale et al., 2015; Frank et al., 2015; Akbari Aghdam et al., 2017; Zheng et al., 2018). The RNA levels of *agg, esp*, and *srtA* markedly decreased when the *E. faecalis* isolates were treated with licochalcone A at 1/4 × MIC for 6 h (Table 2).

**Table 2:** RNA expression levels of biofilm formation-related genes of *E. faecalis* 16C51 and 16C106 isolates with licochalcone A treatment.

**Biofilm formation-related genes**	**16C51**			**16C106**		
	**6 h**	**12 h**	**24 h**	**6 h**	**12 h**	**24 h**
*agg*	0.344	0.690	0.596	0.067	0.749	0.527
*ahrC*	2.235	2.479	1.733	2.071	1.169	0.077
*asa1*	2.028	1.673	0.883	1.030	0.313	0.038
*atn*	2.081	4.683	1.432	2.938	1.429	0.064
*cylA*	1.365	2.685	0.865	0.478	1.563	0.854
*eep*	4.362	9.095	3.752	0.386	0.004	0.492
*ebpA*	0.874	3.654	1.258	1.357	0.687	0.874
*epaI*	1.905	1.634	1.445	1.747	1.126	1.425
*epaOX*	2.046	4.534	8.225	0.547	2.365	1.104
*esp*	0.459	1.269	1.530	0.423	2.181	0.915
*fsrA*	1.025	0.608	0.354	0.936	0.791	0.540
*gelE*	1.354	1.874	0.365	–	–	–
*hyl*	0.874	2.689	1.257	1.068	3.654	0.741
*relA*	0.624	2.354	1.087	1.007	0.698	0.558
*relQ*	1.061	0.841	0.635	0.848	0.903	0.658
*srtA*	0.347	1.568	1.354	0.257	0.874	1.578

Licochalcone A was used at 1/4 × minimum inhibitory concentrations (MICs). RNA levels were detected by RT-qPCR, with untreated isolate as the reference strain (mRNA level = 1.0). –, not detected.

### Genetic mutations in the licochalcone A non-sensitive isolates

**Figure 7:**
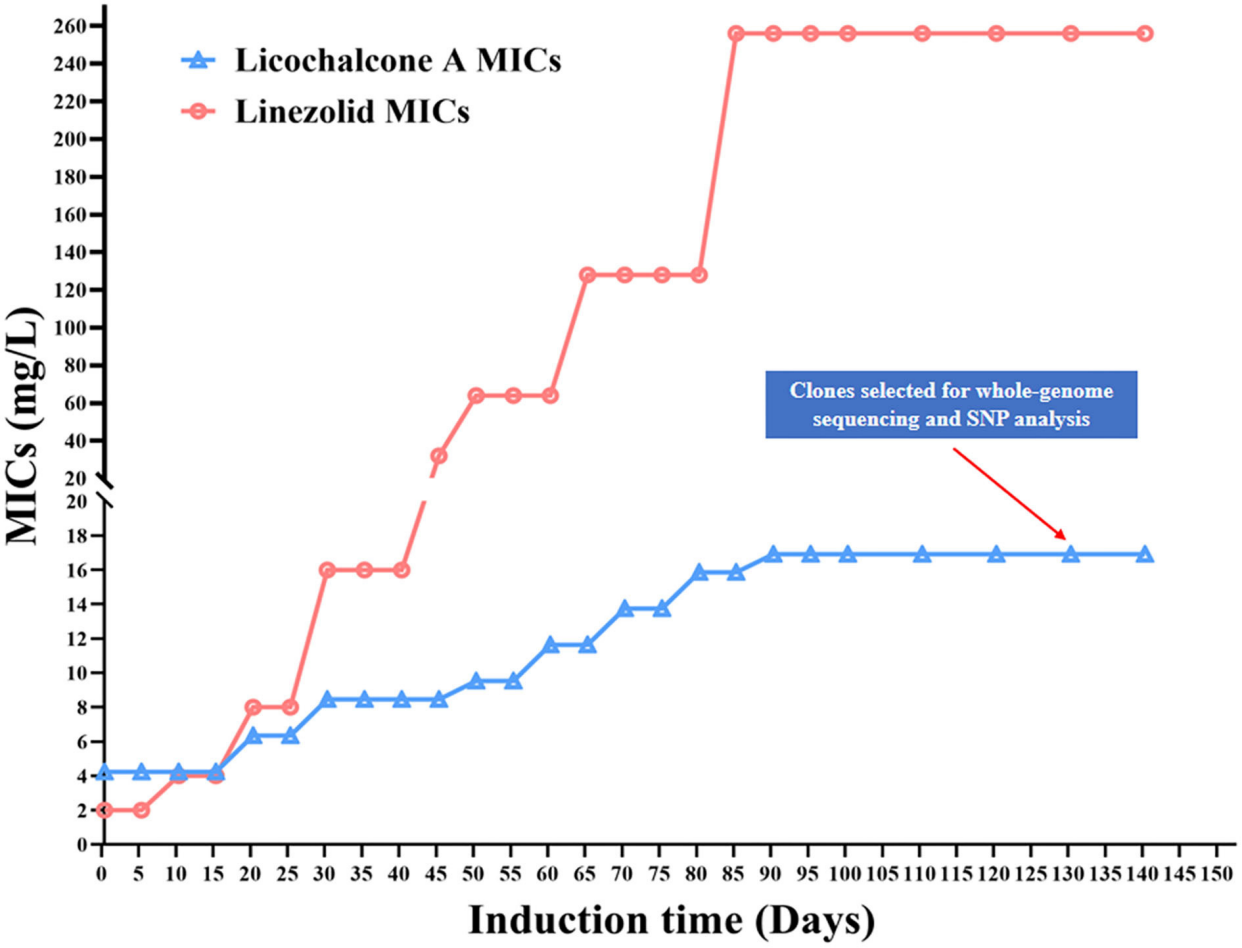
Licochalcone A non-sensitive *E. faecalis* clones induced *in vitro*. The *E. faecalis* 16C51 isolate was serially subcultured in TSB containing licochalcone A or Linezolid, from the initial inducing concentration at 1/2 × MIC, and then successive increased to high concentrations. SNP, Single Nucleotide Polymorphism. MIC, minimum inhibitory concentration.

To investigate the possible target gene of licochalcone A in *E. faecalis*, the licochalcone A non-sensitive *E. faecalis* isolates were selected by *in vitro* induction of wildtype strains under the pressure of licochalcone A, then mutations in the possible target genes were detected by whole-genome sequencing. In the beginning, *E. faecalis* 16C51 and 16C106 isolates were induced, but only the 16C51 isolate with the increased MICs of licochalcone A (50 μM, equal to 16.92 mg/L), which was two times higher than the initial MIC of licochalcone A (25 μM, equal to 8.46 mg/L) after about 140 days of arduous induction (Figure 7). However, as a control, the 16C51 isolate with the increased MICs of linezolid (256 mg/L), which was 64 times higher than the initial MIC of linezolid (4 mg/L) after about 90 days of induction.

Finally, the whole-genomes of the T1 isolate (licochalcone A non-sensitive isolate) and the control isolate (serially subcultured in TSB containing no licochalcone A) from *E. faecalis* 16C51 strain were sequenced by Illumina HiSeq2500. There were 11 nucleotide mutations in the 16C51-T1 isolate leading to nonsynonymous mutations of eight amino acids (Table 3). Among these mutations, there were three mutations located in transcriptional regulator genes (MarR family transcriptional regulator, TetR family transcriptional regulator, and MerR family transcriptional regulator).

**Table 3:** Mutations in *E. faecalis* 16C51-T1 isolate were detected by whole-genome sequencing.

**ref_gene_ID**	**NA mutations**	**AA mutations**	**ref_gene_product**
16C51T1_GM000842	G213T	R70S	MarR family transcriptional regulator
16C51T1_GM001052	T848G	D282A	ethanolamine ammonia-lyase subunit EutC
16C51T1_GM001288	A449C	K149T	TetR family transcriptional regulator
16C51T1_GM001991	C605T	S201F	sigma 54-interacting transcriptional regulator
16C51T1_GM002261	T457C	K152E	helix-turn-helix domain-containing protein
16C51T1_GM002262	T109C	K36E	MerR family transcriptional regulator
16C51T1_GM002375	A65C	E21A	YhgE/Pip domain-containing protein
16C51T1_GM002628	A169G	N56D	DUF3850 domain-containing protein

E. faecalis 16C51 strain was serially subcultured in TSB containing licochalcone A. Mutations in the isolate T1 from 63 generations of 16C51 strain were detected by whole-genome sequencing. NA, nucleotide; AA, amino acid.

## Discussion

Licochalcone A, a novel flavonoid, was extracted from Glycyrrhiza glabra (licorice) and Glycyrrhiza inflata (Chinese licorice; Xue et al., 2018). Licorice is a well-known herb for its unique sweet flavor, now it is utilized to add flavor to foods, beverages, and tobacco, and is widely used as an herbal medicine for gastritis, ulcers, cough, bronchitis, and inflammation (Shen et al., 2019). Up to now, researchers have reported that licochalcone A has antioxidant, anti-angiogenesis, anti-inflammation, and anti-tumor effects (Shen et al., 2019). In recent years, licochalcone A has also been found to have anti-bacterial and anti-viral activities. At present, many studies revealed that licochalcone A had antibacterial activity not only against gram-negative *Salmonella Typhimurium* but also against gram-positive *S. suis, S. aureus*, and *E. faecalis* (Hao et al., 2013; Shen et al., 2015; Grenier et al., 2020; Hosseinzadeh et al., 2020). This study also found that licochalcone A had excellent antibacterial activity against *E. faecalis*. Interestingly, our results demonstrated that licochalcone A could not only rapidly kill *E. faecalis* planktonic cells but also significantly reduced the production of *E. faecalis* persister cells at high concentrations. What's more, this study also found that licochalcone A had a good inhibitory effect on the biofilm formation of *E. faecalis* at sub-inhibitory concentrations.

Licochalcone A indicated a rapid bactericidal effect on *E. faecalis* planktonic cells and was more effective than vancomycin, linezolid, and ampicillin in this study. Ampicillin and vancomycin achieved antibacterial activity by interfering with the cell wall synthesis of Gram-positive bacteria, while linezolid obtained antibacterial activity by interfering with the protein synthesis of Gram-positive bacteria (Suleyman and Zervos, 2016). Therefore, this study suggests that licochalcone A may not through blocking with the cell wall or protein synthesis, but interfere with the intracellular signal transduction or transcriptional regulation against *E. faecalis*. The present study also demonstrated that licochalcone A significantly reduced the production of *E. faecalis* persister cells more than vancomycin, linezolid, and ampicillin at high concentrations. Bacterial persister cells avoid antimicrobials-induced killing by entering physiological dormancy, and it is considered to be one of the main reasons for antimicrobials treatment failure and relapsing infections (Harms et al., 2016). Therefore, reducing the production of bacterial persister cells is expected to reduce chronic or relapsing infections, to improve the effect of anti-infection treatment (Fisher et al., 2017). Licochalcone A isolated from the root of *Glycyrrhiza* species was reported, that had low cytotoxicity against host cells (Si et al., 2018). This means that at high concentrations, licochalcone A may cause less damage to human cells than other chemically synthesized antimicrobials. However, the damaging effect of a high concentration of licochalcone A on host cells still needs to be further studied.

The present study has investigated that licochalcone A significantly inhibited the biofilm formation of *E. faecalis* at sub-inhibitory concentrations. Previous studies also found that licochalcone A inhibited the biofilm formation of *candida albicans, S. suis*, and *S. aureus* (Hao et al., 2013; Shen et al., 2015; Seleem et al., 2016). This study also found that licochalcone A significantly inhibited the transcription of *agg, esp*, and *srtA* in *E. faecalis* during logarithmic growth. The *esp, agg*, and *srtA* are involved in the attachment and early-stage biofilm formation of *E. faecalis* (Tendolkar et al., 2004; Guiton et al., 2009; Kaviar et al., 2022). Therefore, licochalcone A may reduce the biofilm formation of *E. faecalis* by inhibiting its early adhesion and aggregation. Although licochalcone A can significantly reduce the biofilm formation of *E. faecalis*, licochalcone A alone or combined with other antimicrobials has no eradicating effect on the established biofilms of *E. faecalis*. This is similar to other studies which state that once bacterial biofilms are established, it is difficult for antimicrobials to remove them (Shen et al., 2015; Suresh et al., 2019).

To explore the possible target genes of licochalcone A in *E. faecalis*, the licochalcone A non-sensitive *E. faecalis* isolates were selected by *in vitro* induction. Interestingly, this study indicated that *E. faecalis* with the slightly increased MICs of licochalcone A (2 times higher than the initial MIC of licochalcone A) after at least 90 days or 140 days of arduous induction. However, as the control, the same *E. faecalis* isolate with the sharply increased MICs of linezolid (64 times higher than the initial MIC of linezolid) after a similar time of induction as that to licochalcone A. These results suggested that *E. faecalis* was difficult to develop drug resistance under the pressure of licochalcone A, and thus indicates that licochalcone A has a high drug resistance barrier, which is also the potential advantage of its clinical application in future.

Finally, this study detected the gene mutations in the licochalcone A non-sensitive *E. faecalis* isolates by whole-genome sequencing. Our results demonstrated that there were mutations in multiple transcriptional genes (MarR family transcriptional regulator, TetR family transcriptional regulator, and MerR family transcriptional regulator). At present, MarR family transcriptional regulator and TetR family transcriptional regulator were found that closely related to bacterial oxidative stress, biofilm formation, and drug resistance by regulating the efflux pump and signal transduction (Colclough et al., 2019; Fritsch et al., 2019; Nag and Mehra, 2021; Van Loi et al., 2021). Licochalcone A may have multiple target genes in *E. faecalis*, for instance, the MarR family transcriptional regulator and TetR family transcriptional regulator may be the target genes of licochalcone A in *E. faecalis* in this study, which lead to licochalcone A and has a high drug resistance barrier to *E. faecalis*.

The limitation of this study is to find the target gene of licochalcone A in *E. faecalis* through experimental evolution or serial messages. However, this kind of experiment is more valuable to determine the propensity of a given antimicrobial to develop resistance to mutations. Therefore, future research still needs to explore and verify the target of licochalcone A in *E. faecalis* through other methods such as CO-IP and LC-MS.

## Conclusion

In conclusion, this study found that licochalcone A had an antibacterial effect on *E. faecalis* with MIC_50_ and MIC_90_ were 25 μM. Subinhibitory concentrations of licochalcone A significantly inhibited the biofilm formation of *E. faecalis*. MarR family transcriptional regulator and TetR family transcriptional regulator may be the target genes of licochalcone A in *E. faecalis*.

## Data availability statement

The datasets presented in this study can be found in online repositories. The names of the repository/repositories and accession number(s) can be found in the article/Supplementary material.

## Ethics statement

All procedures involving human participants have performed in accordance with the ethical standards of Huazhong University of Science and Technology Union Shenzhen Hospital and with the 1964 Helsinki declaration and its later amendments, and this study was approved by the Ethics Committee of the Huazhong University of Science and Technology Union Shenzhen Hospital.

## Author contributions

XL designed the study, performed biofilm assay, analyzed and interpreted the RNA-seq data, and drafted the manuscript. YX conducted MIC detection, mRNA extraction, RNA-seq, and RT-qPCR data analysis. YS conducted the MIC detection, time-killing test, and biofilm assay. XD and QD performed a biofilm assay, time-killing test, and the *in vitro* licochalcone A induction. YL and ZY analyzed and interpreted the RNA-seq data, the genomic data, and SNP analysis. DL, JZ, and PL designed the study, analyzed the data, and critically revised the manuscript for important intellectual content. All authors contributed to the article and approved the submitted version.

## Funding

This work was supported by the following grants: National Natural Science Foundation of China (82172283); Natural Science Foundation of Guangdong Province, China (2020A1515011049, 2020A1515111146, and 2021A1515011727); Sanming Project of Medicine in Shenzhen (SMGC201705029); Shenzhen Key Medical Discipline Construction Fund (SZXK06162); Science, Technology, and Innovation Commission of Shenzhen Municipality of basic research funds (JCYJ20180302144345028, JCYJ20190809110622729, JCYJ20190809110209389, JCYJ20190809102219774, and JCYJ20190809151817062).

## Conflict of interest

The authors declare that the research was conducted in the absence of any commercial or financial relationships that could be construed as a potential conflict of interest.

## Publisher's note

All claims expressed in this article are solely those of the authors and do not necessarily represent those of their affiliated organizations, or those of the publisher, the editors and the reviewers. Any product that may be evaluated in this article, or claim that may be made by its manufacturer, is not guaranteed or endorsed by the publisher.

## Supplementary material

Supplementary Figure S1 The effect of 8 × MIC of Licochalcone A on the established biofilms of the two *E. faecalis* isolates. The two *E. faecalis* isolates (16C51 and 16C106) formed mature biofilms for 24 h, then treated with Licochalcone A alone, or combined with vancomycin, linezolid, ampicillin at 8 × MIC for 24 h, and the remaining biofilm biomasses were determined by crystal violet staining. The data presented were the average of three independent experiments (mean ± SD). MIC, minimum inhibitory concentration. Lic, licochalcone A; Amp, Ampicillin; Van, Vancomycin; Lin, Linezolid.
