# Supplementary material for: In vitro activities of licochalcone A against planktonic cells and biofilm of Enterococcus faecalis
Source: Front Microbiol. 2022 Oct 21;13:970901. doi: 10.3389/fmicb.2022.970901 (PMC9634178; doi:10.3389/fmicb.2022.970901)
Supplement: Supplementary file 1 [file Table_1.DOCX]

**TABLE S1︱Primers used to detect the biofilm formation related genes by RT-qPCR**.

| **Primers** | **Sequences (5' →3')** | **Location (Genbank no.)** |
| --- | --- | --- |
| *agg*-F | CGTTGATAAAGCAGTTGAT | 52593-52611 (CP002494.1) |
| *agg*-R | TTGTAGTTGGTCTACTTCTT | 52482-52501 (CP002494.1) |
| *ahrC*-F | TTCCATTAGAAACACAAG | 741843-741860 (CP002621.1) |
| *ahrC*-R | GAGAACACTATCATCATC | 742022-742039 (CP002621.1) |
| *asa1*-F | CGCTATTACGAACTATGAC | 3208-3226 (X17214.1) |
| *asa1*-R | GACTTCCAGATACACAGA | 3383-3400 (X17214.1) |
| *atn*-F | AATAATCAATCAGGAACGAATACG | 760647-760670 (NC_004668.1) |
| *atn*-R | GCCACACTAACACCGAAT | 760718-760735 (NC_004668.1) |
| *cylA*-F | GGAGGATATGGTGACAAT | 934-951 (JQ794947.1) |
| *cylA*-R | TTACTTCTGGAGTTGCTAA | 1078-1096 (JQ794947.1) |
| *ebpA*-F | ATAATAACAACGCCATTCAA | 1058348- 1058367 (NC_004668.1) |
| *ebpA*-R | ACATATCACCAGCATCTC | 1058456-1058473 (NC_004668.1) |
| *eep*-F | AACAGATAGAGGCATACC | 1921320-1921337 (CP002621.1) |
| *eep*-R | GCACCACTTATACGATTC | 1921406-1921423 (CP002621.1) |
| *epaI*-F | AGCCGTTCCATCATATTG | 1813792-1813809 (CP002621.1) |
| *epaI*-R | ATGTGACTTCTGGTTATCG | 1813927- 1813945 (CP002621.1) |
| *epaOX*-F | CGTTGAGTAACATTATCGTATTG | 1794588-1794610 (CP002621.1) |
| *epaOX*-R | ATGAAGATATAGTGCCTACCT | 1794715-1794735 (CP002621.1) |
| *esp*-F | GCATCAGTATTAGTTGGT | 172-189 (AF034779.1) |
| *esp*-R | TTCCTTGTAACACATCAC | 350-367 (AF034779.1) |
| *fsrA*-F | GCCTGGATATGATTGTTC | 1591964- 1591981 (CP002621.1) |
| *fsrA*-R | CGTTAGAAGCATTGGTAA | 1592106-1592123 (CP002621.1) |
| *gelE*-F | TACACCATTATCCAGAACT | 1547-1565 (M37185.1) |
| *gelE*-R | CATCGCCATATTGAACTT | 1671-1688 (M37185.1) |
| *hyl*-F | CTTATCTTACCTTAACCAAT | 1336-1355 (AF544400.1) |
| *hyl*-R | CAATTCTGTTCTCAATCTA | 1493-1511 (AF544400.1) |
| *relA*-F | GGATAGATTGATACATATTCG | 1706210-1706230 (CP002621.1) |
| *relA*-R | TGACTTATTAGCCATTCG | 1706338-1706355 (CP002621.1) |
| *relQ*-F | TACGAACGATAGCCACTT | 2157245-2157262 (CP002621.1) |
| *relQ*-R | GCGGATTATGTGCCAATT | 2157356-2157373 (CP002621.1) |
| *srtA*-F | CGTGTTGAGTTAATTGATGA | 2930172-2930191 (NC_004668.1) |
| *srtA*-R | TTGCTGCTAATGTTCCTT | 2930263-2930280 (NC_004668.1) |
| recA-F**^a^** | CGACTAATGTCTCAAGCACTAC | 3044995-3045016 (NC_004668.1) |
| recA-R | CGAACATCACGCCAACTT | 3044911-3044928 (NC_004668.1) |

**^a^**: The internal control gene was recA (OG1RF_12439)
