# Supplementary figures and images for: In vitro activities of licochalcone A against planktonic cells and biofilm of Enterococcus faecalis
Source: Front Microbiol. 2022 Oct 21;13:970901. doi: 10.3389/fmicb.2022.970901 (PMC9634178; doi:10.3389/fmicb.2022.970901)

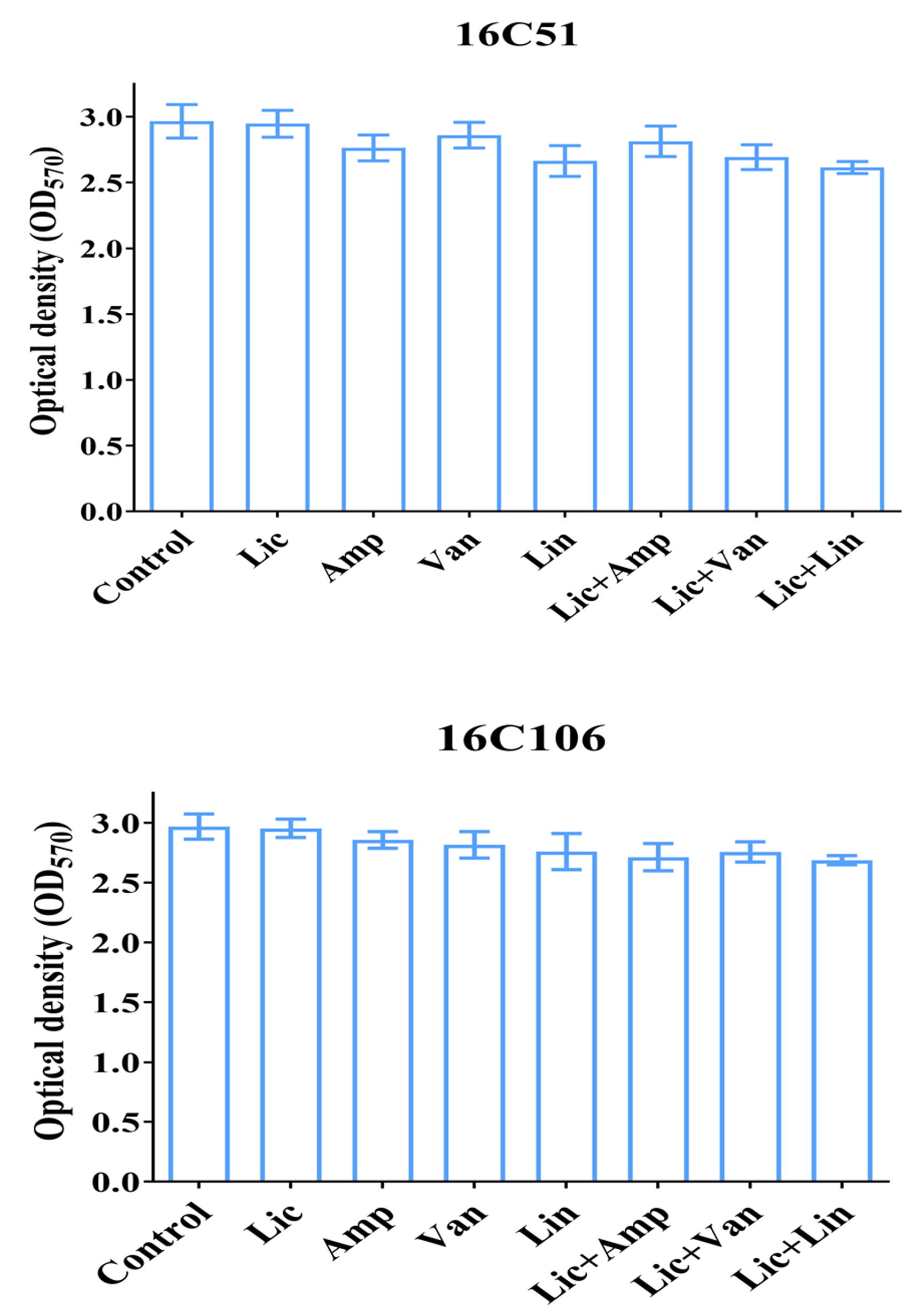

Supplement: Supplementary Figure S1 — The effect of 8 × MIC of Licochalcone A on the established biofilms of the two E. faecalis isolates. The two E. faecalis isolates (16C51 and 16C106) formed mature biofilms for 24 h, then treated with Licochalcone A alone, or combined with vancomycin, linezolid, ampicillin at 8 × MIC for 24 h, and the remaining biofilm biomasses were determined by crystal violet staining. The data presented were the average of three independent experiments (mean ± SD). MIC, minimum inhibitory concentration. Lic, licochalcone A; Amp, Ampicillin; Van, Vancomycin; Lin, Linezolid. [file Image_1.TIF]
